# Prevalence and Risk Factors of Pain Among Older People in Mexico: Evidence From MHAS 2018

**DOI:** 10.1155/jare/5547878

**Published:** 2026-03-27

**Authors:** Tianwei Zhang, Qumin Chen, Xiaodong Li, Baoshan Hu

**Affiliations:** ^1^ Department of Anesthesiology, The First Affiliated Hospital of Xiamen University, School of Medicine, Xiamen University, Xiamen, 361003, China, xmu.edu.cn; ^2^ Department of Obstetrics and Gynecology, The First Affiliated Hospital of Xiamen University, School of Medicine, Xiamen University, Xiamen, 361003, China, xmu.edu.cn; ^3^ Department of Orthopedics, The First Affiliated Hospital of Xiamen University, School of Medicine, Xiamen University, Xiamen, 361003, China, xmu.edu.cn

**Keywords:** MHAS, older people, pain, prevalence, risk factors

## Abstract

**Objective:**

To explore the prevalence of pain and its risk factors among older people in Mexico.

**Methods:**

We conducted a cross‐sectional analysis using data from the 2018 Mexican Health and Aging Study (MHAS), which included 8958 participants aged 60 years and above. Variables including pain, social demographics, and health‐related factors were collected through questionnaire surveys, and a binary logistic regression model was used to identify risk factors associated with pain.

**Results:**

The prevalence of pain among older people in Mexico was 40.8%. Multivariate regression analysis showed that female gender, obesity, retirement, incapacity or inability to work, hypertension, diabetes, arthritis/rheumatism, heart attack, and cancer history were positively correlated with pain. In contrast, higher levels of education, widowhood, household chores, and exercising more than three times a week are negatively correlated with pain. However, age, smoking, and alcohol consumption did not show any significant correlation to pain.

**Conclusion:**

The prevalence of pain is relatively high among older people in Mexico. Targeted intervention measures should be taken to improve awareness of the risk factors and the prevention of pain.

## 1. Introduction

Pain is a global public health issue. In a survey conducted on adults aged 25 years and above from 52 countries, the prevalence of pain ranged from 9.9% to 50.3%, and the older people, women, and rural residents were affected the most [1]. The prevalence of pain in the general adult population is related to factors such as female gender, advanced age, low education level, smoking, alcohol consumption, central obesity, emotional disorders, and sedentary behavior [2]. Among the older people, pain is more prevalent in women with less education and worse economic conditions and can be alleviated through physical activity [3]. Hypertension, diabetes, arthritis, and other diseases are also risk factors for pain [4].

A 6‐year longitudinal data study using the National Health and Aging Trends Study (NHATS) showed that approximately 50% of older people suffer from long‐term persistent pain, and over 25% suffer from long‐term activity restriction pain. The prevalence of both types of pain is continuously increasing worldwide [5]. Persistent pain can lead to a decline in physical function and happiness among older people within 7 years [6]. In addition, pain can impair their reactive balance and increase the risk of fall‐related fractures [7]. The prevalence of disability in patients with chronic pain is as high as 51.5% [8]. In Norway, the annual cost of chronic pain accounts for 4% of GDP, with 80% of the cost being due to loss of productivity [9]. Therefore, active measures need to be taken to alleviate pain among older people.

With a rapidly aging global population, the proportion of older people aged 60 years and above in Mexico is expected to increase from 6.3% in 2010 to 23% in 2050 [10]. The prevalence of pain among older people in Mexico at the end of their lives is 71.5% [11], although the available research on pain‐related factors is limited. Mexican clinicians lack sufficient understanding of and adherence to pain management guidelines [12], and only 36% of Mexican adults in need of pain treatment have access to opioid medications, which is merely 1% of the amount obtained in the United States [13]. These findings indicate a significant gap in pain management in Mexico compared to developed countries. The aim of this study was to explore the prevalence and risk factors of pain in older people in Mexico.

## 2. Methods

### 2.1. Study Population

The Mexican Health and Aging Study (MHAS) is a large, longitudinal, family‐based cohort study of older people (aged ≥ 50 years) and their spouses in Mexico, and the data are representative of the national population [14]. In this study, we used the data of MHAS 2018, which included a total of 17114 respondents. The response rate for the 2018 MHAS survey was 85%. After excluding respondents younger than 60 years and those with missing data, 8958 participants were included in the study. The MHAS study was approved by the National Institutes of Health/National Institute on Aging (grant number: NIH R01AG018016) and the National Institute of Statistics and Geography (INEGI) of Mexico. All participants provided informed consent. The data were obtained from the MHAS, which is a publicly available and anonymized database. As this study involved a secondary analysis of de‐identified data, it was exempt from institutional review board approval. The study design is shown in Figure 1.

**FIGURE 1:**
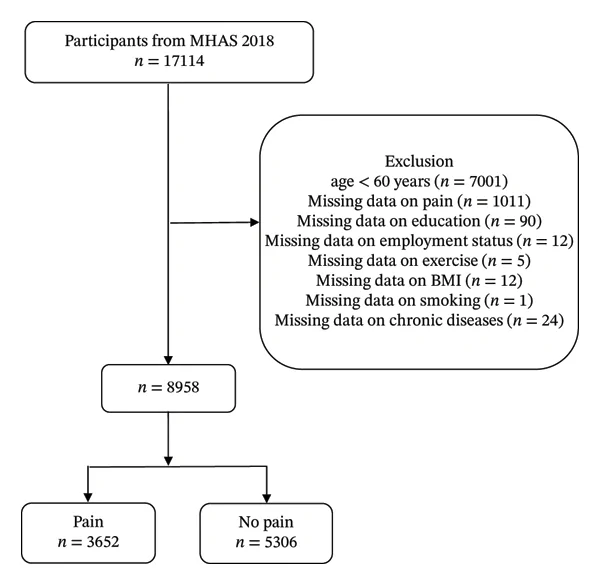
The flowchart of population screening.

### 2.2. Dependent Variable

The dependent variable was determined based on self‐reported pain assessment in MHAS. Participants were asked, “Do you often suffer from pain?” (yes/no), and the “yes” response was indicative of pain.

### 2.3. Independent Variables

Sociodemographic and health‐related factors were the independent variables. Sociodemographic characteristics included age (60–69, 70–79, and ≥ 80 years), gender (male or female), marital status (married or in civil union, separated or divorced, widowed, and single), education level (no education, 1–5, 6, 7–9, and over 10 years of education), and employment status (currently working, currently looking for a job, household chores, retired, or incapacitated). The health‐related factors were as follows: exercising more than 3 times a week, current smoking, current alcohol consumption, body mass index (BMI), and combined chronic diseases (diabetes, hypertension, heart attack, arthritis/rheumatism, and cancer). The presence of chronic diseases was ascertained by the response to the question “Have doctors or healthcare professionals diagnosed you with (specific chronic disease)?” The subjects were classified as underweight (< 18.5 kg/m^2^), normal weight (18.5–24.9 kg/m^2^), overweight (25.0–29.9 kg/m^2^), or obese (≥ 30.0 kg/m^2^) according to their BMI [15]. Due to the small number of underweight individuals, they were included in the normal weight group [16].

### 2.4. Statistical Analysis

Statistical analysis was performed using SPSS 27.0 software (IBM Corporation, Armonk, NY, USA). Categorical variables were represented by numbers and percentages. The chi‐square test was used to compare categorical variables between the pain group and the non‐pain group. Multivariate binary logistic regression was used to identify the risk factors for pain, and the Hosmer and Lemeshow test was used to evaluate the goodness of fit of the model. The variables with statistical significance (*p* < 0.05) in the univariate analysis were included in the multivariate logistic regression analysis, and the odds ratio (OR) and corresponding 95% confidence interval (CI) were calculated. *p* < 0.05 was considered statistically significant.

## 3. Results

During the 2018 MHAS cycle, 56.2% of the participants were female, 45.5% were aged between 60 and 69 years, and 43.1% were overweight. Most of the respondents had received some level of education (82.1%), were married (62.6%), did not smoke (90.2%), did not drink alcohol (76.3%), exercised less than 3 times a week (71%), were retired, and had lost the ability to work or did not work (40%). The chronic diseases recorded in the population were hypertension (51.5%), diabetes (27.9%), arthritis/rheumatism (14.9%), heart attack (4.7%), and cancer (3%). The prevalence of pain was 40.8% (Table 1).

**TABLE 1:** Baseline characteristics of the study population.

Characteristic	Total	No pain	Pain	*p* value
	*n* (%)	*n* (%)	*n* (%)	
*Total N*	8958 (100)	5306 (59.2)	3652 (40.8)	
*Age (years)*				0.001
60–69	4075 (45.5)	2465 (46.5)	1610 (44.1)	
70–79	3489 (38.9)	2075 (39.1)	1414 (38.7)	
≥ 80	1394 (15.6)	766 (14.4)	628 (17.2)	

*Gender*				< 0.001
Male	3923 (43.8)	2651 (50.0)	1272 (34.8)	
Female	5035 (56.2)	2655 (50.0)	2380 (65.2)	

*BMI (kg/m^2^)*				< 0.001
< 25	3047 (34.0)	1924 (36.3)	1123 (30.8)	
25.0–29.9	3865 (43.1)	2353 (44.3)	1512 (41.4)	
≥ 30	2046 (22.8)	1029 (19.4)	1017 (27.8)	

*Educational level (years)*				< 0.001
No education	1603 (17.9)	843 (15.9)	760 (20.8)	
1–5	2854 (31.9)	1633 (30.8)	1221 (33.4)	
6	1969 (22.0)	1172 (22.1)	797 (21.8)	
7–9	1403 (15.7)	864 (16.3)	539 (14.8)	
≥ 10	1129 (12.6)	794 (15.0)	335 (9.2)	

*Marital status*				0.002
Married or in a civil union	5606 (62.6)	3391 (63.9)	2215 (60.7)	
Separated or divorced	637 (7.1)	377 (7.1)	260 (7.1)	
Widowed	2302 (25.7)	1287 (24.3)	1015 (27.8)	
Single	413 (4.6)	251 (4.7)	162 (4.4)	

*Employment status*				< 0.001
Currently working	2590 (28.9)	1696 (32.0)	894 (24.5)	
Currently looking for work	140 (1.6)	87 (1.6)	53 (1.5%)	
Household chores	2649 (29.6)	1534 (28.9)	1115 (30.5)	
Retired, incapacitated, or does not work	3579 (40.0)	1989 (37.5)	1590 (43.5)	

*Hypertension*				< 0.001
No	4346 (48.5)	2908 (54.8)	1438 (39.4)	
Yes	4612 (51.5)	2398 (45.2)	2214 (60.6)	

*Diabetes*				< 0.001
No	6459 (72.1)	3961 (74.7)	2498 (68.4)	
Yes	2499 (27.9)	1345 (25.3)	1154 (31.6)	

*Arthritis/rheumatism*				< 0.001
No	7627 (85.1)	4887 (92.1)	2740 (75.0)	
Yes	1331 (14.9)	419 (7.9)	912 (25.0)	

*Heart attack*				< 0.001
No	8536 (95.3)	5119 (96.5)	3417 (93.6)	
Yes	422 (4.7)	187 (3.5)	235 (6.4)	

*Cancer*				< 0.001
No	8690 (97.0)	5179 (97.6)	3511 (96.1)	
Yes	268 (3.0)	127 (2.4)	141 (3.9)	

*Exercise 3* *+* *times per week*				< 0.001
No	6364 (71.0)	3621 (68.2)	2743 (75.1)	
Yes	2594 (29.0)	1685 (31.8)	909 (24.9)	

*Currently drinks alcohol*				< 0.001
No	6834 (76.3)	3969 (74.8)	2865 (78.5)	
Yes	2124 (23.7)	1337 (25.2)	787 (21.5)	

*Currently smokes tobacco*				< 0.001
No	8079 (90.2)	4734 (89.2)	3345 (91.6)	
Yes	879 (9.8)	572 (10.8)	307 (8.4)	

The risk factors associated with pain are summarized in Table 2. Univariate analysis showed that age, gender, BMI, education level, marital status, employment status, hypertension, diabetes, arthritis/rheumatism, heart attack, cancer, exercising frequency, smoking, and drinking were significantly related to pain (*p* < 0.05). The *p* value calculated by the Hosmer and Lemeshow test was 0.724, indicating a good fit of the model. The results of the multiple logistic regression analysis (Table 2) showed that gender, obesity, retirement, and disability were positively correlated with pain. Women were 1.9 times more likely to experience pain than men (OR = 1.890, 95% CI = 1.678–2.130, *p* < 0.001). The prevalence of pain in obese individuals (OR = 1.501, 95% CI = 1.326–1.699, *p* < 0.001) was higher compared to that in individuals with normal weight. The risk of pain among those who were retired, had lost their ability to work, or were not working was 1.2 times higher than those who were currently working (OR = 1.206, 95% CI = 1.069–1.361, *p* = 0.002). Furthermore, hypertension (OR = 1.480, 95% CI = 1.347–1.627, *p* < 0.001), diabetes (OR = 1.151, 95% CI = 1.040–1.274, *p* = 0.007), arthritis/rheumatism (OR = 3.397, 95% CI = 2.986–3.865, *p* < 0.001), heart attack (OR = 1.727, 95% CI = 1.400–2.130, *p* < 0.001), and cancer (OR = 1.441, 95% CI = 1.112–1.868, *p* = 0.006) were associated with a significantly higher risk of pain. In contrast, higher levels of education, widowhood, household chores, and exercising more than three times a week were negatively correlated with pain, whereas age, smoking, and alcohol consumption did not show any significant correlation.

**TABLE 2:** Pain‐related factors identified from univariate and multivariate logistic regression models.

Characteristic	Univariate analysis	*p* value	Multivariate analysis	*p* value
	or (95% CI)		or (95% CI)	
*Age (years)*				
60–69	Reference		Reference	
70–79	1.043 (0.951–1.144)	0.368	0.923 (0.832–1.025)	0.133
≥ 80	1.255 (1.110–1.419)	< 0.001	1.050 (0.904–1.220)	0.520

*Gender*				
Male	Reference		Reference	
Female	1.868 (1.713–2.038)	< 0.001	1.890 (1.678–2.130)	< 0.001

*BMI(kg/m^2^)*				
< 25	Reference		Reference	
25.0–29.9	1.101 (0.998–1.214)	0.054	1.073 (0.966–1.191)	0.187
≥ 30	1.693 (1.511–1.897)	< 0.001	1.501 (1.326–1.699)	< 0.001

*Educational level (years)*				
No education	Reference		Reference	
1–5	0.829 (0.733–0.938)	0.003	0.818 (0.718–0.932)	0.002
6	0.754 (0.660–0.862)	< 0.001	0.751 (0.651–0.866)	< 0.001
7–9	0.692 (0.598–0.800)	< 0.001	0.634 (0.541–0.742)	< 0.001
≥ 10	0.468 (0.398–0.550)	< 0.001	0.493 (0.414–0.588)	< 0.001

*Marital status*				
Married or in a civil union	Reference		Reference	
Separated or divorced	1.056 (0.894–1.247)	0.523	0.872 (0.727–1.045)	0.137
Widowed	1.207 (1.094–1.332)	< 0.001	0.828 (0.738–0.930)	0.001
Single	0.988 (0.805–1.212)	0.909	0.867 (0.695–1.080)	0.203

*Employment status*				
Currently working	Reference		Reference	
Currently looking for work	1.156 (0.814–1.642)	0.419	1.123 (0.776–1.625)	0.540
Household chores	1.379 (1.233–1.542)	< 0.001	0.746 (0.649–0.857)	< 0.001
Retired, incapacitated, or does not work	1.517 (1.366–1.683)	< 0.001	1.206 (1.069–1.361)	0.002

*Hypertension*				
No	Reference		Reference	
Yes	1.867 (1.714–2.034)	< 0.001	1.480 (1.347–1.627)	< 0.001

*Diabetes*				
No	Reference		Reference	
Yes	1.360 (1.239–1.493)	< 0.001	1.151 (1.040–1.274)	0.007

*Arthritis/rheumatism*				
No	Reference		Reference	
Yes	3.882 (3.427–4.398)	< 0.001	3.397 (2.986–3.865)	< 0.001

*Heart attack*				
No	Reference		Reference	
Yes	1.883 (1.546–2.292)	< 0.001	1.727 (1.400–2.130)	< 0.001

*Cancer*				
No	Reference		Reference	
Yes	1.638 (1.284–2.089)	< 0.001	1.441 (1.112–1.868)	0.006

*Exercise 3* *+* *times per week*				
No	Reference		Reference	
Yes	0.712 (0.648–0.783)	< 0.001	0.888 (0.800–0.986)	0.026

*Currently drinks alcohol*				
No	Reference		Reference	
Yes	0.815 (0.738–0.902)	< 0.001	1.068 (0.954–1.194)	0.253

*Currently smokes tobacco*				
No	Reference		Reference	
Yes	0.760 (0.657–0.879)	< 0.001	1.066 (0.911–1.247)	0.426

## 4. Discussion

The results of our cross‐sectional study on the MHAS 2018 dataset indicate a high prevalence (40.8%) of pain among the older people in Mexico. Consistent with this, 39.6% of the population aged 60 years and above in India [17] and about 37.8% individuals aged 65 and above in the United States [18] experience pain. Among Mexican Americans over 67 years of age, the incidence of self‐reported pain is 34.8% [19], which is slightly lower compared to our findings. A study from 15 European countries showed that the prevalence of pain in adults over 50 years of age was between 30% and 60% [20]. These differences can be attributed to race, cultural factors, and the use of pain assessment scales.

In our study cohort, female gender, obesity, retirement, loss of work ability, or inability to work were positively correlated with pain. Among the older people, women are more susceptible to chronic pain compared to men, and the risk correlates positively with obesity [21]. In addition, women are more sensitive to pain and experience more pain‐related illnesses than men [22]. A study conducted in China also reported a higher risk of pain among women in the population aged 45 years and above [23]. High BMI is a major risk factor for pain among older people [24], and obesity is associated with increased severity of pain and high disability rates [25]. A nationwide cross‐sectional study in Portugal reported that older adults, retirees, unemployed individuals, and those with a lower education level were at an increased risk of developing pain [26]. Likewise, older people in the United States who were unable to work due to disabilities are more likely to report chronic pain [18], which is consistent with the results of this study.

Multivariate regression analysis further identified hypertension, diabetes, arthritis/rheumatism, heart attack, and cancer history as the risk factors of pain among older people. About 66.1% of patients aged 50 years and above in the United States suffer from at least five chronic diseases [27], and the number of chronic diseases correlates positively with the degree of pain [28]. In developed countries, hypertension and pain are correlated at the population level. For instance, a cross‐sectional study in Saudi Arabia showed a significant correlation between hypertension and chronic pain [29], and the prevalence of pain is significantly higher among hypertension patients aged 65 years and above in the United States [30]. Both diabetes and prediabetes are significantly associated with chronic pain symptoms [31]. In addition, diabetic neuropathy, rheumatoid arthritis, and osteoarthritis are closely related to neuropathic pain, which is common in middle‐aged and older people and significantly impacts their health [32]. Chronic pain in older people aged 65 years and above in the United States is significantly correlated with osteoarthritis, rheumatism, and peripheral neuropathy, which is consistent with the results of this study [33]. Furthermore, chronic pain may increase the risk of myocardial infarction, heart failure, and cardiovascular death [34]. Studies show that the overall prevalence of pain in cancer patients is 49.4%, and the prevalence of neuropathic pain is 36.2%, which significantly impairs the patients’ quality of life [35]. A South Korean study identified female gender, history of ischemic heart disease, knee arthritis, and cancer as the risk factors for chronic back pain in adults [36].

In line with our findings, a cross‐sectional study conducted across the United States, United Kingdom, China, and India showed that people with higher levels of education have a lower risk of pain and less interference with daily activities [37]. A negative correlation between education level and pain has been reported for older people in Thailand [38], and higher education level is an established protective factor for pain [39]. A sedentary lifestyle and lack of exercise are risk factors for chronic musculoskeletal pain, whereas mild to moderate intensity physical exercise two to three times a week can mitigate pain associated with chronic diseases [40]. Furthermore, regular sport activities can reduce the risk of new or worsening pain in older people [41] and increase the probability of recovery for moderate to severe chronic pain [42]. In this study, the prevalence of pain was lower among those engaged in household chores, which may be related to their nonsedentary lifestyle. Research has shown that older age, longer duration of physical labor, and lack of physical activity are all associated with a greater number of painful areas [43]. In this study, engaging in household chores was negatively correlated with pain, which may be related to moderate physical activity. A study based on MHAS examining the trajectory of activity limiting pain found that marriage is associated with increased pain among Mexican older people aged 50 and over [44]. Among the three groups of divorced, widowed, and single individuals in this study, only widowed individuals were associated with reduced pain, indicating that the relationship between marital status and pain warrants further investigation. On the other hand, age, smoking, and alcohol consumption were not significantly correlated with pain. A cross‐sectional survey in China yielded similar results, showing no significant correlation between age and chronic pain in individuals aged 60 years and above [45]. However, previous studies have identified smoking and alcohol consumption as risk factors for pain [23], thereby warranting further research.

This study has several advantages, including the use of a nationally representative sample of older people in Mexico, which enhances the generalizability of the findings. Although Mexico has made progress in pain management, a significant gap remains between management policies and practices. The lack of sufficient infrastructure and professional personnel as well as substantial barriers to the prescription of opioid drugs ,have seriously hindered the implementation of management policies [46]. Therefore, pain management policies and practices in Mexico require further strengthening.

This study has some limitations that ought to be addressed. First, we could not establish a causal relationship between pain and any of the risk factors due to the cross‐sectional design of the study. Second, pain assessment was conducted through questionnaire surveys, and the results may have been biased due to differences in pain sensitivity and subjectivity. In addition, there was no detailed classification or evaluation of pain type, duration, or severity. Third, we only focused on the older people in Mexico, and the results may not be applicable to older people in other countries.

## 5. Conclusion

Pain is prevalent among the older people in Mexico, and its risk factors include female gender, obesity, retirement, incapacity or inability to work, hypertension, diabetes, arthritis/rheumatism, heart attack, and cancer history. Moderate physical exercise and a higher level of education are protective factors against pain. These factors should be included in public health intervention plans to develop targeted prevention strategies and pain management measures. Future research should incorporate longitudinal, nationally representative data and more detailed pain assessments to better understand pain among older people in Mexico.

## Author Contributions

Qumin Chen: downloaded and organized the data. Xiaodong Li and Baoshan Hu: methodology and validation. Tianwei Zhang: statistical analysis of the data. Baoshan Hu and Tianwei Zhang: wrote the manuscript.

## Funding

No funding was received for this study.

## Conflicts of Interest

The authors declare no conflicts of interest.

## Data Availability

The data presented in this study are available on request from the corresponding author.
